# Ultrasound-guided decompression surgery of the distal tarsal tunnel: a novel technique for the distal tarsal tunnel syndrome—part III

**DOI:** 10.1007/s00276-019-02196-w

**Published:** 2019-02-23

**Authors:** Simone Moroni, Alejandro Fernández Gibello, Marit Zwierzina, Gabriel Camunas Nieves, Rubén Montes, José Sañudo, Teresa Vazquez, Marko Konschake

**Affiliations:** 1 Department of Podiatry, Faculty of Health Sciences at Manresa, Universitat de Vic-Universitat Central de Catalunya (UVic-Ucc), Barcelona, Spain; 2 Clinic Vitruvio Biomecánica, Madrid, Spain; 3 Department of Podiatry, Faculty of Health Sciences, University of La Salle, Madrid, Spain; 4 Department of Plastic, Reconstructive and Aesthetic Surgery, Center of Operative Medicine, Medical University of Innsbruck, Innsbruck, Austria; 5 Division of Clinical and Functional Anatomy, Department of Anatomy, Histology and Embryology, Medical University of Innsbruck (MUI), Müllerstr. 59, 6020 Innsbruck, Austria; 6 Anatomy and Embryology Department, School of Medicine, Complutense University of Madrid, Madrid, Spain

**Keywords:** Tarsal tunnel, Heel pain, Ultrasound, Minimally invasive, Ultrasound-guided, Nerve entrapment

## Abstract

**Background:**

The aim of this study was to provide a safe ultrasound-guided minimally invasive surgical approach for a distal tarsal tunnel release concerning nerve entrapments.

**Methods and results:**

The study was carried out on ten fresh-frozen feet. All of them have been examined by high-resolution ultrasound at the distal tarsal tunnel. The surgical approach has been marked throughout the course of the medial intermuscular septum (MIS, the lateral fascia of the abductor hallucis muscle). After the previous steps, nerve decompression was carried out through a MIS release through a 2.5 mm (± 0.5 mm) surgical portal. As a result, an effective release of the MIS has been obtained in all fresh-frozen feet.

**Conclusion:**

The results of our anatomic study indicate that this novel ultrasound-guided minimally invasive surgical approach for the release of the MIS might be an effective, safe and quick decompression technique treating selected patients with a distal tarsal tunnel syndrome.

## Introduction

Since the tarsal tunnel syndrome (TTS) was first described by Keck et al. [17] and by Lam et al. [22] in the early 60s, foot and ankle practitioners have evaluated many ways of treating it surgically if non-operative treatments failed [12, 28].

The true incidence of the TTS is unknown [7], but a specific cause can be identified in 60–80% of the patients [18, 24], nevertheless, the literature states a 50% idiopathic cause of the TTS [41].

In those cases, in which space-occupying mass or systemic disorders, except diabetes, are not present, the etiology for a TTS has been hypothesized as being a fibrosis and/or thickening of osteofibrous structures at this critical anatomic region [32].

In a diabetic foot, thickening and degeneration of the tibial nerve (TN) and its branches are present [32]. Thus, reducing the space within the tarsal tunnel leads to an entrapment of the TN and/or its branches and increases the risk of sensorimotor neuropathy and diabetic foot complications [31].

Different studies show that in the chronic plantar heel pain syndrome, a focal entrapment of a single TN branch or a direct, more proximal entrapment of the TN can be found in the tarsal tunnel in 88%; this exists as a single entity or also in association with other causes [28, 36].

**Fig. 1:**
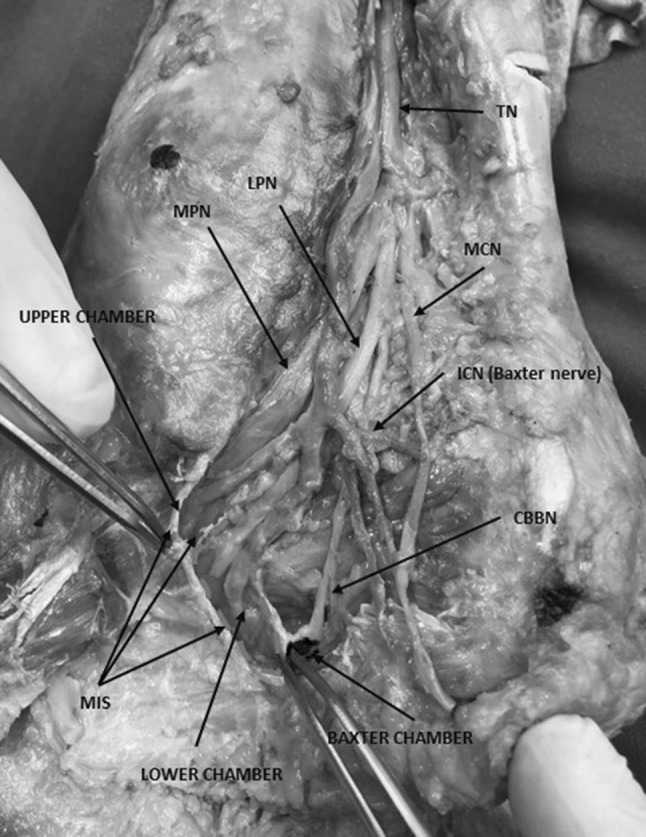
Overview of the main components of the proximal and distal tarsal tunnel and all important chambers. *TN* tibial nerve, *mpn* medial plantar nerve, *lpn* lateral plantar nerve; *icn* inferior calcaneal nerve (i.e., baxter nerve), *cbbn* calcaneal branch of the baxter nerve, *mcn* medial calcaneal nerve, *ms* medial septum, *upper chamber* blue rounded area bounded by blue dotted line, *lower chamber* green rounded area bounded by green dotted line; “Baxter chamber”: red rounded area bounded by red dotted line. (Color figure online)

Heimkes et al. already in 1987 published a paper, which highlighted osteofibrous structures in the tarsal tunnel in detail [14]. He found two different regions where the tibial nerve (TN) could be compressed by osteofibrous structures within the tunnels [14]: in the proximal region (the “proximal tarsal tunnel”), the nerve could be entrapped beside the flexor retinaculum which leads to a proximal tarsal tunnel syndrome; the second region was named the “distal tarsal tunnel” [14]. While, as mentioned, for the proximal tarsal tunnel syndrome, the compression is caused by the flexor retinaculum, within the distal tarsal tunnel, two possible fibrous structures might be important: the medial intermuscular septum (MIS) of the sole of the foot (= deep fascia of the abductor hallucis muscle) [39], which should be considered as the thicker medial “wall” of the distal tarsal tunnel; and second its triangular extension, the medial intermuscular septum extension described by Heimkes et al. as a connective tissue partition that originates from the medial side of the calcaneus or the fibrous sheath of the flexor hallucis longus and inserts on the lateral aspect of the MIS. The medial intermuscular septum extension forms a middle bridge for two osteofibrous tubes in which the TN branches are running through [14]. It is also called “interfascicular septum”, which divides the main branches of the TN running through a superior and inferior calcaneal tube [11, 29]. Figure 1 shows an overview of the main components of the proximal and distal tarsal tunnel (Fig. 1).

These fibrous structures in the distal tarsal tunnel might be crucial for the development of a distal tarsal tunnel syndrome [14, 29]. Already in 2009, it could be demonstrated that to reduce the pathological pressure at the TN and its branches in this area, it is crucial to release these fibrous structures, as well as a release of the flexor retinaculum [37].

Most of the surgeons state in literature performing a proximal tarsal tunnel release by open surgery using also an extension of the procedure to the distal tarsal tunnel to get better outcomes, trying to widen the narrow spaces caused by those thickened and inextensible fascial structures in this area [2, 13, 20, 43].

However, the majority of the surgeons who described these open tarsal tunnel decompression surgery techniques only recommend a release of the flexor retinaculum for the proximal and the MIS for the distal tarsal tunnel, advocating good overall outcomes [1, 2, 12, 13, 21–23, 28, 30, 37, 38, 40, 44].

Over the years, open decompression techniques for the distal tarsal tunnel release associated to a proximal tarsal tunnel release have been described [14, 33]. In the last decade, some authors also described minimally invasive release via endoscopy for an isolated nerve entrapment of the TN branches [6, 21, 25].

Nevertheless, until now, after publishing our part II study of a proximal tarsal tunnel release technique [8, 11, 29], there was no surgical procedure published about an ultrasound-guided, ultra-minimally invasive technique with a release of the distal tarsal tunnel, which can also be included in the cluster of the ultrasound-guided foot and ankle decompression surgery (UGAFDS) technique.

Therefore, the aim of this study part III was to describe and prove the effectiveness and safety of an UGAFDS technique for a distal tarsal tunnel release technique for selected patients suffering from a distal tarsal tunnel syndrome.

## Materials and methods

For this study, an ultrasound-guided surgical approach on ten fresh-frozen feet (six male, four female) was performed. The individuals had given their written informed consent prior to death for their use for scientific and educational purposes and donated their bodies to the University Complutense of Madrid (Center of Body Donation). According to National Law, scientific institutions (in general Institutes, Departments or Divisions of Medical Universities) are entitled to receive the body after death mainly by means of a specific legacy, which is a special form of last will and testament. No bequests are accepted without the donor having registered their legacy and been given appropriate information upon which to make a decision based upon written informed consent (policy of ethics) [19, 27]; therefore an ethics committee approval was not necessary [19, 27].

Exclusion criteria of the cadavers were: BMI above 30 (impaired ultrasound echogenicity), signs of traumas in the ankle region, a history of ankle or foot ischemic vascular disorders, surgery or space-occupying mass lesions.

### Materials

**Fig. 2:**
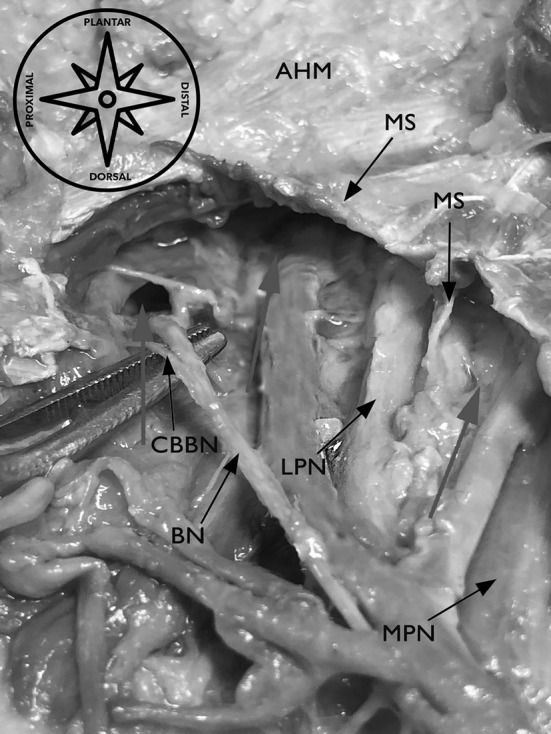
Nerves (lpn, mpn, bn) entering separated tubes at the distal tarsal tunnel, perforating the medial septum. *AHM* abductor hallucis muscle, *mpn* medial plantar nerve, *lpn* lateral plantar nerve, *bn* Baxter’s nerve, *cbbn* calcaneal branch of the Baxter’s nerve, *ms* medial septum and its extension, *red arrows* nerves entering upper and lower calcaneal tubes

**Fig. 3:**
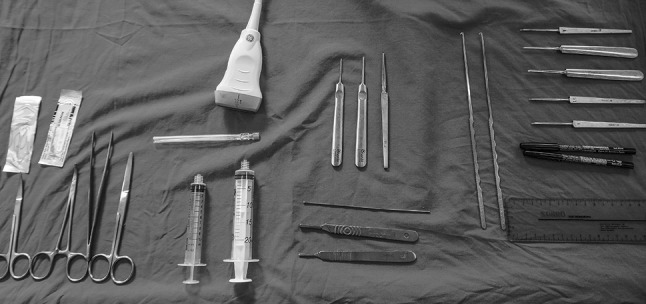
Instruments for the minimally invasive ultrasound-guided procedure. High-resolution ultrasound; dissection material; 20-gauge needle; syringe; V-shape; hooked knife

**Fig. 4:**
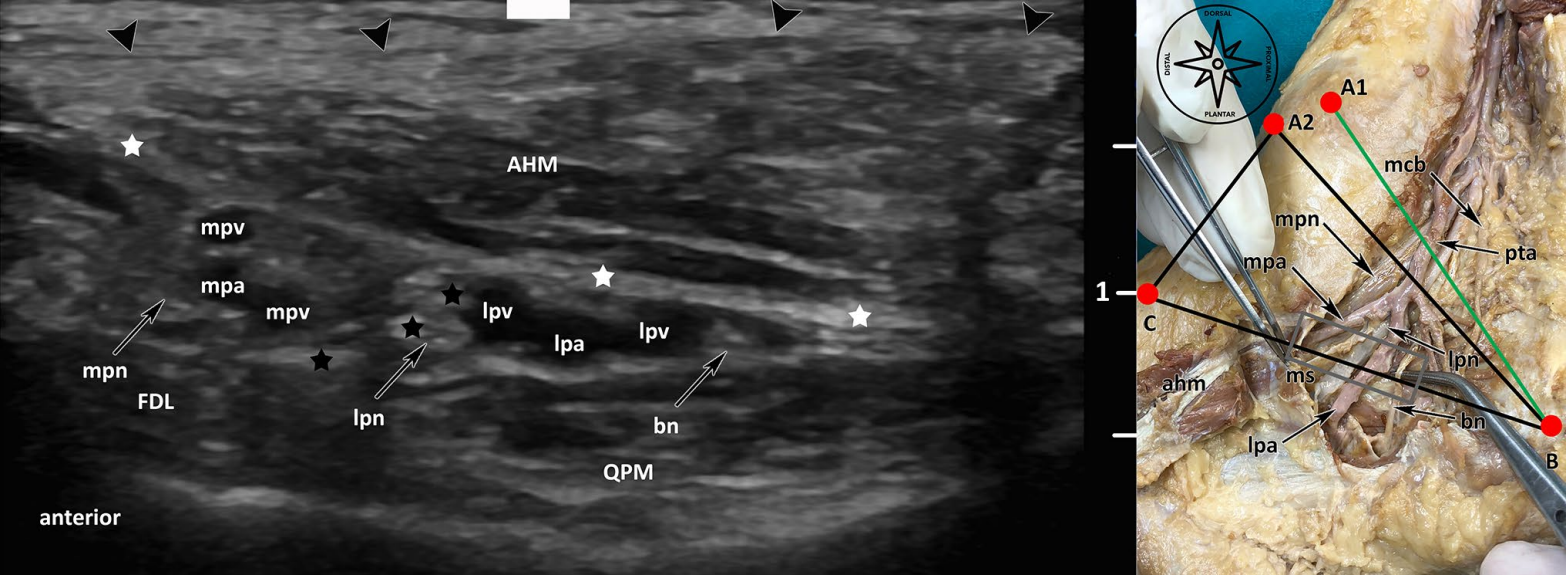
Ultrasound visualization of the terminal branches (lpn, mpn, bn) on the [BC] line. The figure on the right side shows an anatomical overview of the structures including the reference line “Dellon-McKinnon” (A_1_–B, malleolar-calcaneal) and the “Triangle of Heimkes” (A_2_–B–C). *Lpn* lateral plantar nerve, *mpn* medial plantar nerve, *bn* Baxter’s Nerve, *FHL* flexor hallucis longus muscle, *QPM* quadratus plantae muscle, *AHM* abductor hallucis muscle, *mpv* medial plantar vein, *lpv* lateral plantar vein, *mpa* medial plantar artery, *lpa* lateral plantar artery, *black arrow heads* superficial layer of the flexor retinaculum, *white stars* medial septum (deep fascia of the abductor hallucis muscle), *black stars* medial septum extension (“interfascicular septum”)

The equipment used performing the minimally invasive ultrasound-guided decompression procedure for the distal tarsal tunnel release were as follows (Figs. 1, 2, 3, 4): high-frequency ultrasound with 17Mhz linear probe (Sonoscape, Italy, p-40), 20-gauge needle and a syringe of 10 cc, V-shape gouges of 1, 2 mm 5 cm length, a hooked knife and a buttoned probe.

The surgical procedures were performed by two podiatric surgeons with more than 5 years of experience in ultrasound-guided procedures guided by a clinical anatomist, also trained in clinical anatomy and surgery for years.

### Relevant US anatomy of the distal tarsal tunnel and foot position (Figs. 1, 2, 3, 4)

The specimens were positioned in a decubitus supinatus, dorsiflexed-everted position (“frog leg position”) for all surgical procedures.

The sonoanatomy of this region has been evaluated in detail before the surgery in all ten specimens.

At the “BC line”, taken from our part I of our study series, we found from medial to lateral [29]: a subcutaneous tissue with a typical lobular fat of variable echogenicity with the mean thickness of 0.5 cm (± 0.3 cm), the tiny medial band of the plantar fascia (superficial abductor hallucis muscle fascia) which envelopes the abductor hallucis muscle and appears as an hyperechoic linear slice. The abductor hallucis belly has a variable cross-sectional area (mean 1 cm ± 0.3 cm). The structure that envelops the abductor hallucis belly laterally is the thick medial intermuscular septum (MIS, the lateral fascia of the abductor hallucis muscle), which appears as a ligamentous hyperechoic structure with a mean thickness of 0.9 cm (± 0.1 cm). From this fascial layer, right in the middle, one can recognize the interfascicular septum, which appears as a hyperechoic band, inserting in most of the cases at the flexor hallucis longus fibrous sheet with a great variability in thickness (mean 0.08 cm ± 0.3 cm) dividing the upper tube dorsally from the lower tube plantarly.

Laterally and slightly plantarly, one can recognize the less hyperechoic quadratus plantae muscle fascia, in the same plane, just medially to the bony hyperechoic landmark (the sustentaculum tali), and one can see the flexor hallucis longus muscle in the short axis, right lateral to the MIS insertion and plantarly to the sustentaculum tali as an echoic dotted ovular shape structure with a mean thickness of 0.6 cm (± 0.1 cm). Dorsally to the MIS (i.e., in the upper tube), one can see the most dorsal structure, the flexor digitorum longus tendon in the short axis (main thickness 0.5 cm ± 0.1 cm) as an echoic dotted ovular shape structure, the medial plantar nerve in the short axis as an honey-hive echoic structure with a mean width of 0.3 cm (± 0.07 cm) and the medial plantar vascular bundle composed by the artery and mostly two veins as hypoechoic circular structures. All of those structures were embedded in a variable echoic fatty tissue. Plantarly to the MIS, one can recognize the most dorsal structure, the lateral plantar nerve in the short axis also as an honey-hive echoic structure with a mean width of 0.3 cm (± 0.04 cm); the lateral plantar vascular bundle as hypoechoic circular structures and the “Baxter nerve” (in literature also known as the first branch of the lateral plantar nerve, anterior branch of the calcaneal nerve or the inferior calcaneal nerve [4, 16, 35]) in the short axis as an hypoechoic, “monofascicular” nerve with a mean width of 0.15 cm (± 0.05 cm) **(**Fig. 4**)**.

### Step-by-step approach of the ultrasound decompression surgery for the distal tarsal tunnel syndrome

#### Step I

**Fig. 5:**
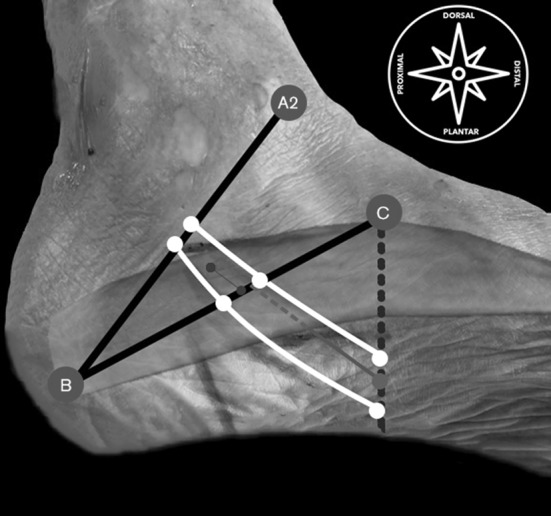
Presurgical mapping of the distal tarsal tunnel. A2-B line: a line from the anterior tip of the medial malleolus (anterior colliculus) to the center of the posterior calcaneal tuberosity; CB line: a line from the navicular tuberosity to the center of the posterior calcaneal tuberosity. *Red dots* from C perpendicular to the sole of the foot: reference for the surgical portal; *white dots* and *lines*: course of MPN and LPN through A2-B, BC and C projection lines; *blue dots* and *line*: skin projection of medial septum extension from its intersection with BC line and its mean proximal point calculated from our anatomical study part I; *muscle belly:* represents the full dorso-plantar height of the medial intermuscular septum; *red line*: surgical portal for the medial intermuscular septum release in the upper calcaneal tube; dotted tract of the surgical portal line represents the full cut length for the medial intermuscular septum release. (Color figure online)

The first step, using a surgical pen, was to draw the osseous references for the BC line and the A2–B line [29] (Figs. 4, 5).

#### Step II

The second step was to mark the lateral plantar nerve (LPN) and its first branch (Baxter nerve) and the medial plantar nerve (MPN) where they crossed the BC line under ultrasound guidance in the short axis. To follow the course of the nerves, by elevator technique, we marked the point where MPN and LPN crossed the imaginary lines from point C, perpendicular to the planta pedis. We marked the point where the nerves crossed this line representing the anatomical courses of the nerves (Figs. 4, 5).

#### Step III

The third step was to draw the landmarks for the MIS under US guidance; we marked on the skin the point at the BC line and 1 cm proximal [29] (Fig. 5).

#### Step IV

The fifth and last presurgical step was to get a safe surgical portal between the previous drawn lines, which represented the courses of the MPN and the LPN, slightly distal to the perpendicular line to the planta pedis passing through point C. The surgical line portal and the safe working space are now drawn on the skin from this last point and the most proximal point for the interfascicular septum (Fig. 5).

### Surgical technique

#### Step V

The next step consisted of introducing a 20-gauge traumatic needle previously curved at 90º at the distal point of our “virtual surgical portal” previously drawn. With the 20-g needle, we performed the US-guided hydrodissection making sure that the tip of the needle stays beneath the MIS to make sure that we increased the “working space” at the surgical line, moving away the medial and lateral plantar neurovascular bundles. Once the tip of the needle reached the proximal point of our surgical line, we stopped hydro-dissecting and left the needle under the surgical line for an ultrasound “guide”.

#### Step VI

Using the needle as a guide, under continued US guidance, we introduced the V-shape gouge of 1 mm, followed by the V-shape gouge of 2 mm from the same portal to enlarge the “working space” to insert the hooked knife.

#### Step VII

The last step for the distal tarsal tunnel release was as follows: after creating a safe channel that allowed us to introduce a 3 mm hooked knife using a 2 mm V-shape gouge, beneath the MIS, we kept particular attention to maintain the hook parallel to the MIS and with the cut looking to the interfascicular septum, then turning it perpendicular with the “cutting edge” towards the MIS. Then we retired the 2 mm V-shape, carefully introduced the retrograde knife through the surgical portal with the blade rotated towards the MIS until the proximal portal point, thus we did a retrograde cut under US guidance of the superior and inferior calcaneal chambers pushing it “towards” the skin to avoid damage to nerves or vascular structures. Before retiring the retrograde knife, we introduced the buttoned probe all the way through the surgical line, pushing it towards the skin to ascertain that the MIS was cut entirely. If not, step VIII was repeated again.

The buttoned probe was left inside all the way through the surgical line to get the proof of the line for the instruments used during the entire procedure.

**Fig. 6:**
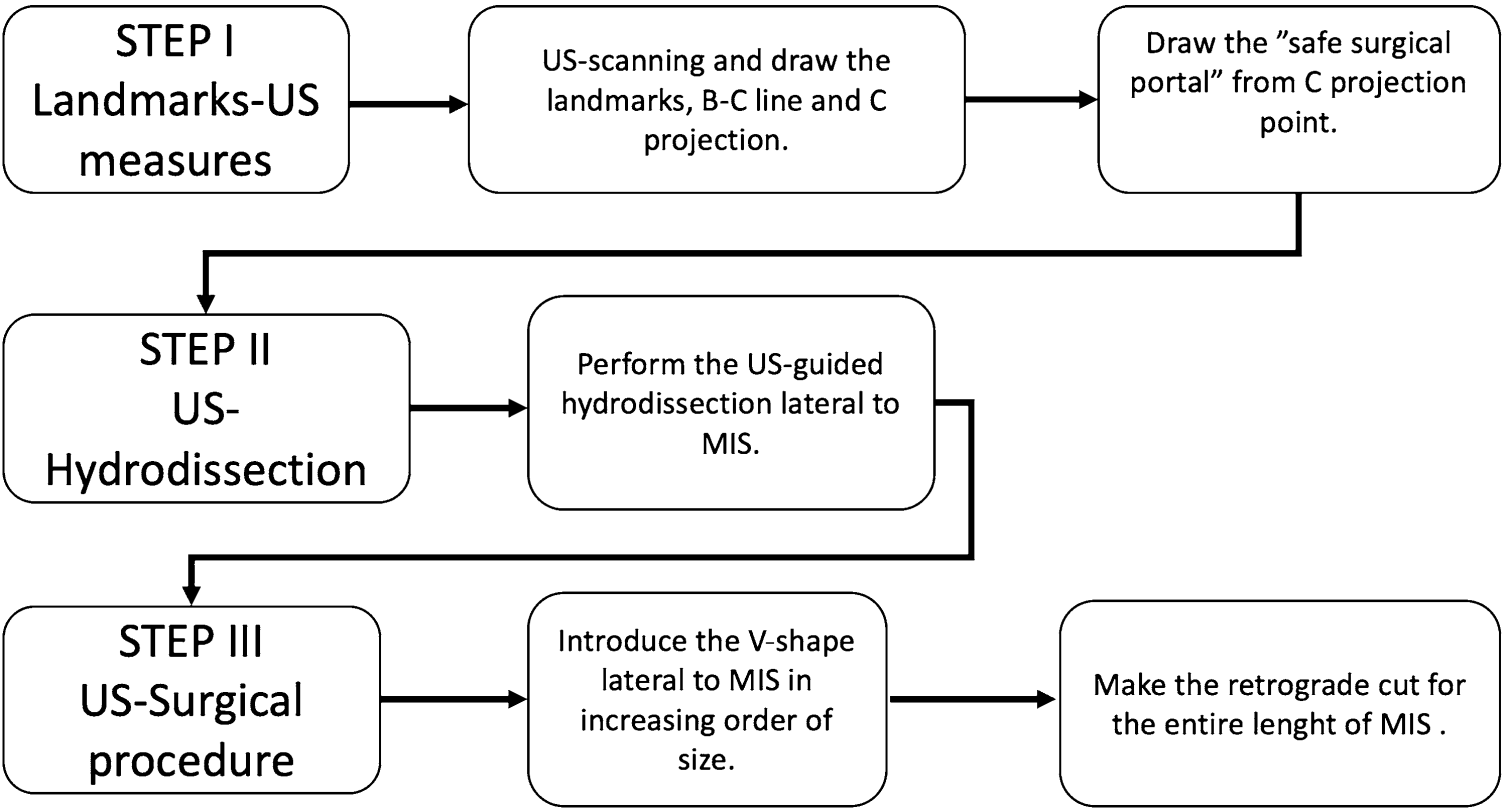
Algorithm. *BC-line* a line from the navicular tuberosity to the center of the posterior calcaneal tuberosity, *C projection* perpendicular line from the navicular tuberosity to the sole of the foot, *MIS* medial intermuscular septum

All these steps were performed under continued US guidance (Fig. 6).

### Postsurgical anatomical findings

After the procedure was completed, the clinical anatomist dissected the ten feet. The dissection started from the medial ankle region to the medial aspect of the sole of the foot to ascertain the effectiveness and safety of the technique. The skin and the subcutaneous fat were cut off, the medial band of the plantar fascia, overlying the abductor hallucis muscle (AHM) was incised, and the muscle belly of the AHM was retracted to expose the portion of the MIS above the superior and inferior calcaneal chambers, to ascertain if it was completely released. Moreover, all the deep structures in relation to the MIS such as the medial and lateral neurovascular plantar bundles were intended to be preserved to verify every damage to those structures during the UGAFDS technique in the superior and inferior calcaneal tubes.

## Results

The average duration of the procedure, including all VII steps, within our cluster of ten feet was approximately 18 min (± 4 min), decreasing in time for each procedure due to the learning curve, from 35 min for the first procedure to 14 min for the last one.

In all ten feet, osseus landmarks were clearly identified, despite four out of ten feet having a BMI between 25 and 30.

For the presurgical scanning of the tibial nerve in all ten subjects, the TN branches (medial and lateral plantar nerve, Baxter´s nerve, medial calcaneal branch) were identified and the localization spots were drawn at the BC line (step II) [29] and distally at the imaginary line passing for C, perpendicular to the sole of the foot (step V) to get a representation of the topographical distribution of the TN branches on the skin at the distal tarsal tunnel [29]. In all ten feet, it was possible to visualize the interfascicular septum at both references points (Step III).

For the surgical steps, the ultrasound-guided procedures were used to perform the surgical gesture, to avoid damage to all the neurovascular structures. All instruments could be visualized well under US guidance during the steps VI and VII.

**Fig. 7:**
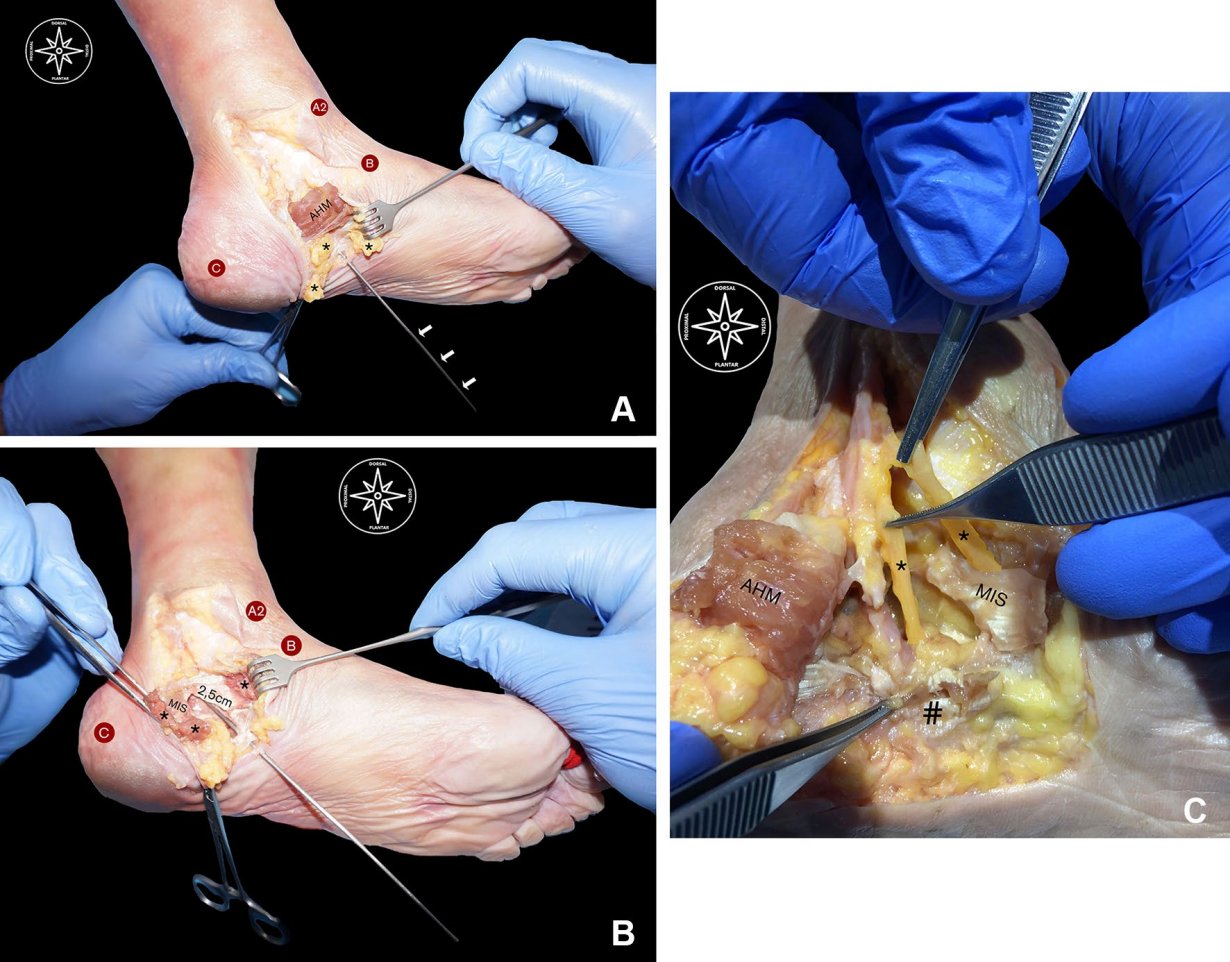
**a** Dissection routine. A2: medial malleolus B: navicular tuberosity C: center of the posterior calcaneal tuberosity; *AHM*: exposed abductor hallucis muscle belly; *asterisk* subcutaneus fat; *white arrows*: buttoned probe entering the surgical portal and following the course through the surgical line. **b** Gross anatomical findings. *A2* medial malleolus; *B* navicular tuberosity; *C* center of the posterior calcaneal tuberosity; *asterisk* abductor hallucis muscle belly over the medial intermuscular septum; 2.5 cm: the mean length of the retrograde cut performed over the MIS which exposes the buttoned probe entering the surgical portal and following the course through the surgical line. **c** Gross anatomical findings. MIS: medial intermuscular septum; *AHM* abductor hallucis muscle belly over the medial intermuscular septum; *numbers sign* plantar fascia; *asterisk* proof of undamaged medial and lateral plantar nerves. (Color figure online)

After the procedure for every foot has been concluded, the clinical anatomist started calculating the mean incision length for the surgical portal. The mean length of the incision was 2.5 mm (± 0.5 mm). Then, after meticulous dissection, the effectiveness of the retrograde cut towards the MIS in the superior and inferior calcaneal chambers was evaluated: the cut was made successfully for the entire length (mean cut length 2.5 cm ± 0.5 cm) of the MIS in the superior as well as in the inferior calcaneal chambers (Fig. 7 a, b).

### Complications

Within the ten cadaveric feet, neither in the superior nor in the inferior calcaneal chamber, a neurovascular plantar structure was damaged (Fig. 7c).

## Discussion

The plantar heel pain syndrome affects almost 10% of adults in their lifetime. In the USA, between 1 and 2 million of patients require a treatment for this issue each year [10, 33, 34]. It has been hypothesized that up to 20% of the plantar heel pain syndrome, mimicking plantar fasciopathy symptoms could be caused due to an entrapment beneath the MIS.

Zheng et al. showed that almost 5% of patients, diagnosed with radiculopathy (L4-L5-S1), also suffer from a tarsal tunnel syndrome [45].

At the same time, it has been seen that up to 30% of the diabetic population suffer from diabetic sensorimotor polyneuropathy (DSNP), with typical stocking-glove symptoms in the hands and feet [5]. In the USA, 22.3 million of people suffer from diabetes and 3–6% of them have the risk of developing a diabetic foot ulcer each year [3, 5].

Based on the results of Tekin et al. concerning ultrasonographic evaluation of hemodynamic changes in patients with DSNP after tarsal tunnel decompression, it can be suggested that nerve release procedures have also a positive effect on the hemodynamic and morphological parameters of the arteries, as they pass through the anatomical tunnels nearby the nerves as well as positive effects on the neurological functions of the entrapped nerves [40].

Moreover in diabetic patients, Trignano et al. found an improvement of the peripheral microcirculation in the diabetic foot after surgical decompression [42]. This might be an important point for patients with chronic ulcers [42].

At the foot and ankle region, Heimkes, Dellon and Kumar in their studies defined the greatest risk site for the entrapment of the TN branches at the distal tarsal tunnel underneath the MIS and beside the interfascicular septum [14, 37, 39].

Thus, the distal tarsal tunnel remains a challenging anatomical region for podiatric physicians treating those two prevalent conditions, plantar heel pain syndrome and painful DSNP that affects a great number of patients each year [3, 42].

Rosson et al. demonstrated that performing a neurolysis at the distal tarsal tunnel in addition to a flexor retinaculum release lowers the overall tarsal tunnel pressure, compared to a proximal tarsal tunnel release alone in patients with a tarsal tunnel syndrome [37].

Lundborg et al. demonstrated that an increased pressure over a nerve for a prolonged period of time could lead to a reduced interfascicular vasa nervorum (vas nutritium) flow constituting an important pathophysiological mechanism and explaining nerve pain and damage beneath osteofibrous structures [26].

The literature also shows that an open surgical release of both at the proximal and distal tarsal tunnel produced good outcomes in association to a partial plantar fasciotomy for patients suffering from recalcitrant plantar heel pain syndrome [28].

Other encouraging data for open surgical outcomes were described by Watson et al. for a distal tarsal tunnel release suggesting that open surgical procedures in association to partial plantar fasciotomy may improve outcomes in selected patients [43].

Hendrix et al. found a 96% rate of positive outcomes in treating intractable plantar heel pain syndrome with a release of all fibrous retaining systems for the plantar nerves like the TN, the medial and lateral plantar and the Baxter’s nerve [15]. Extensive tarsal tunnel decompression for painful diabetic patients with DSNP has been first discussed by Dellon et al. in “A cause for optimism in diabetic neuropathy” in 1988, claiming that this procedure could reduce neuropathy-related symptoms [9].

Already in 1992, a prospective study showed that decompression surgery in patients with DSNP improves sensorimotor function in 85% of the patients, which already confirms the hypothesis that nerve compression plays an important role in diabetic neuropathy [9].

For the last 30 years, Dellon’s technique for a tarsal tunnel release has been the most used procedure among foot and ankle surgeons for patients suffering from DSNP getting promising results [9]. In this study, Dellon reported that 73% of the patients showed an improvement in pain after a 12-month follow-up.

Concerning applied surgical techniques, it has been seen that minimally invasive surgery for a tarsal tunnel release revealed the similar outcomes compared to open releases [28], at the same time minimizing soft tissue dissection, potential wound complications like infections and scar fibrotization with reducing time recovery and avoiding offloading [40].

Our ultra-minimally invasive procedure presented in this study could be therefore performed at the distal tarsal tunnel with maximizing effects of the open procedure described by Dellon et al. due to a surgical access of 2.5 mm only.

Of course, more future, clinical surgical studies are necessary in the near future applying our novel technique of an ultrasound-guided ankle and foot decompression surgery (UGAFDS) at the proximal and distal tarsal tunnels in selected patients suffering from DSNP and recalcitrant plantar heel pain syndrome to further evaluate the effectiveness of these procedures.

## Conclusions

The results of our clinical-anatomic study indicate that this novel ultrasound-guided ankle and foot decompression surgery (UGAFDS) technique for a minimally invasive release of the medial intermuscular septum (the lateral fascia of the abductor hallucis muscle) might be an effective, safe and quick decompression technique for treating selected patients with a distal tarsal tunnel syndrome.
